# Retraction: MicroRNA-20a-5p promotes colorectal cancer invasion and metastasis by downregulating Smad4

**DOI:** 10.18632/oncotarget.28669

**Published:** 2024-11-07

**Authors:** Dantong Cheng, Senlin Zhao, Huamei Tang, Dongyuan Zhang, Hongcheng Sun, Fudong Yu, Weiliang Jiang, Ben Yue, Jingtao Wang, Meng Zhang, Yang Yu, Xisheng Liu, Xiaofeng Sun, Zongguang Zhou, Xuebin Qin, Xin Zhang, Dongwang Yan, Yugang Wen, Zhihai Peng

**Affiliations:** ^1^Department of General Surgery, Shanghai General Hospital, School of Medicine, Shanghai Jiao Tong University, Shanghai, China; ^2^Department of Pathology, Shanghai General Hospital, School of Medicine, Shanghai Jiao Tong University, Shanghai, China; ^3^Department of Pathology, Fudan University Affiliated Shanghai Cancer Center, Shanghai, China; ^4^Department of Gastroenterology, Shanghai General Hospital, School of Medicine, Shanghai Jiaotong University, Shanghai, China; ^5^Department of Oncology and Department of Clinical and Experimental Medicine, Linköping University, Linköping, Sweden; ^6^Department of Gastrointestinal Surgery, West China Hospital, Sichuan University, Chengdu, Sichuan, China; ^7^Department of Neuroscience, School of Medicine, Temple University, Philadelphia, PA, USA; ^8^Department of Pathology, Zhejiang Provincial People’s Hospital, Hangzhou, Zhejiang, China; ^*^These authors contributed equally to this work

**This article has been retracted:** In Figure 5D, the same western blot image was used for both the HT29 and HCT116 cell groups. The authors are unable to make corrections using the original data. As a result, the Editorial decision was made to retract this paper. All authors agreed with the decision. The Shanghai Jiao Tong University authorities were notified about the issues in this paper.

Original article: Oncotarget. 2016; 7:45199–45213. 45199-45213. https://doi.org/10.18632/oncotarget.9900

